# Enterobius Vermicularis infection of the appendix as a cause of acute appendicitis in a Greek adolescent: a case report

**DOI:** 10.1186/1757-1626-1-376

**Published:** 2008-12-06

**Authors:** Eleni Efraimidou, Anthia Gatopoulou, Charilaos Stamos, Nikolaos Lirantzopoulos, George Kouklakis

**Affiliations:** 1 1^st ^Surgical Department, Democritus University of Thrace, Alexandroupolis, Greece; 2 Endoscopy Unit, Democritus University of Thrace, Alexandroupolis, Greece; 3 Department of pathology, Democritus University of Thrace, Alexandroupolis, Greece

## Abstract

Gastrointestinal infection due to Enterobius vermicularis occurs worldwide and is considered to be the most common helminth infection. The simple presence of E. vermicularis in the appendix usually produces symptoms of acute appendicitis. The association of this parasitic infestation with acute appendicitis varies from 0.2%–41.8% worldwide. We present a case of a 15 year old female with enterobiasis of appendix presented with clinical features of acute appendicitis. The appendix was surgically removed and the specimen was pathologically diagnosed to contain of E. vermicularis in non-inflamed and histologically normal appendix. Even if this condition is not uncommon in the Greek population, to the best of our knowledge this is the first report presented in the English literature.

## Introduction

The presence of parasites in the appendix may cause appendiceal colic even without eliciting an acute inflammation. This colic due to a parasitic infestation is explained by the hypothesis of appendiceal lumen obstruction. [1]

Gastrointestinal infection due to Enterobius vermicularis occurs worldwide and is considered to be the most common helminthes infection. [1] The simple presence of E. vermicularis in the appendix usually produces symptoms which resemble acute appendicitis although the mechanism for this does not involve mucosal invasion by the parasite. [2] We present a case of enterobiasis of appendix presented with clinical features of acute appendicitis. Even if this condition is not uncommon in the Greek population, to the best of our knowledge this is the first report presented in the English literature.

## Case Presentation

A 15 years old female (student, 48 kg weight, 162 cm height) presented initially with diffuse acute abdominal pain. Within 12 hours of onset, she noted anorexia without nausea or vomiting. The patient's abdominal pain typically increased in intensity and the characteristic shift in the pain to the right lower quadrant occurred. She reported mild fever. Her laboratory findings included only an elevated total white blood count of 11.000/mm3 without elevated proportion of eosinophils, whereas all other tests were within normal ranges, included ultrasound examination.

Right lower quadrant tenderness and rigidy were found on abdominal palpation. Rovsing's sign and examination for rectal tenderness was also positive. Due to the combination of the classic symptoms and a typical progression of symptoms coupled with right lower quadrant tenderness, acute appendicitis was suspected and surgical removal was decided.

**Figure 1:**
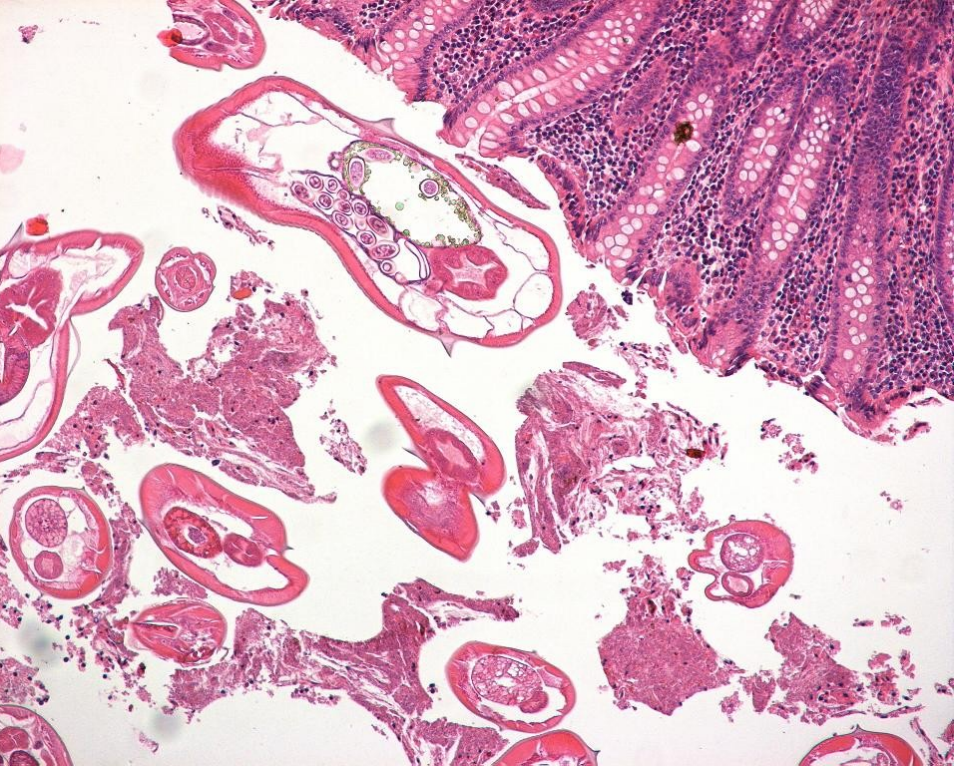
Helminthes in the lumina.

At pathological examination, macroscopically normal appendix was noted. This was confirmed histopathologically. The lumen contained parasites with features compatible with Enterobius vermicularis. (Figure 1). The diagnosis was parasitic infestation in surgical removed appendices. After the recovery, mebendazole for the affected and for all the family members was prescribed. A single dose of 100 mg was recommended and the patient received a second treatment after 15 days.

## Discussion

Enterobius vermicularis is an extremely well adapted parasite that usually produces no specific symptoms in most colonized persons. Most symptoms are minor such as pruritus ani and restless sleeping. People of every socioeconomic group may acquire pinworm infection and it remains quite prevalent. [3]

A variety in geography is noted. [1] While often considered tropical, parasitic diseases are now seen more frequently in development countries because of immigration and increased world travel. There was no travel history in our case.

The association between parasitic infection of the appendix and acute appendicitis has been widely investigated. [4] Retrospective studies have indicated that Enterobius vermicularis is the commonest worm found in the appendix and that its presence can cause pathologic changes ranging from lymphoid hyperplasia to acute phlegmonous inflammation with life-threatening complications like gangrene and peritonitis.[4]

A retrospective analysis conducted in Turkish population found among 190 appendectomies, 6 specimens (3, 15%) to contain parasites, 4 of them were Enterobius vermicularis. [1] Similar percentage (3.8%) reported other studies from Turkey, as well. [5,6] In Nepal Enterobius vermicularis was identified in 1.62% among patients with clinical diagnosis of appendicitis. [2]

In one Brazilian study, 24 cases out of 1600 appendectomies (1.5%) with helminthes within the appendix were recorded during a 10-year period [4]

Finally, one recent study from Iran confirmed the relationship between Enterobius vermicularis and the occurrence of acute appendicitis in 2.9% cases. [7]

Slightly higher incidence (4%) was described in another study from Denmark which also concluded that there was a highly significant difference in the incidence of Enterobius vermicularis in normal appendices and in inflamed which may indicate that the presence of this parasite in the appendix can give the symptoms of acute appendicitis or this parasite leaves or does not enter an inflamed appendix. [8]

In our case, absence of histological inflammation and macroscopically normal appearance of the appendix were observed. A histological audit of acute appendicitis in the past confirmed this association. [9]

Moreover, there are reports that suggest presence of Enterobius infestations with acute appendicitis, ruptured appendicitis, or with no significant clinical symptoms in children. [10]

Unfortunately, there are no studies conducted in Greece, therefore we ignore the incidence of Enterobius infestations in Greek population. Even if acute appendicitis due to Enterobius vermicularis is not uncommon in Greece, to the best of our knowledge, this is the first report in Greek population.

In conclusion, the presence of Enterobius vermicularis usually accounts for appendiceal related pain in the absence of histological inflammation. High index of suspicion and including parasitic origin in differential diagnosis of abdominal disturbances might hopefully prevent unnecessary surgeries.

## Consent

"Written informed consent was obtained from the patient and parents for publication of this case report and accompanying image. A copy of the written consent is available for review by the Editor-in Chief of this journal"

## Competing interests

The authors declare that they have no competing interests.

## Authors' contributions

Dr. EF performed the surgery and revised the manuscript. Dr. GA was a major contributor in writing the manuscript and analyzed, interpreted the data. Dr. CS performed the histological examination and gave final approval. Dr. NL performed the surgery and gave the final approval. Dr. GK gave final approval and revised the article.
